# Sensitivity and Validity of Psychometric Tests for Assessing Driving Impairment: Effects of Sleep Deprivation

**DOI:** 10.1371/journal.pone.0117045

**Published:** 2015-02-10

**Authors:** Stefan Jongen, Joy Perrier, Eric F. Vuurman, Johannes G. Ramaekers, Annemiek Vermeeren

**Affiliations:** 1 Department of Neuropsychology and Psychopharmacology, Faculty of Psychology and Neuroscience, Maastricht University, Maastricht, The Netherlands; 2 U 1075 COMETE, INSERM, Caen, France; University of California, San Francisco, UNITED STATES

## Abstract

**Objective:**

To assess drug induced driving impairment, initial screening is needed. However, no consensus has been reached about which initial screening tools have to be used. The present study aims to determine the ability of a battery of psychometric tests to detect performance impairing effects of clinically relevant levels of drowsiness as induced by one night of sleep deprivation.

**Methods:**

Twenty four healthy volunteers participated in a 2-period crossover study in which the highway driving test was conducted twice: once after normal sleep and once after one night of sleep deprivation. The psychometric tests were conducted on 4 occasions: once after normal sleep (at 11 am) and three times during a single night of sleep deprivation (at 1 am, 5 am, and 11 am).

**Results:**

On-the-road driving performance was significantly impaired after sleep deprivation, as measured by an increase in Standard Deviation of Lateral Position (SDLP) of 3.1 cm compared to performance after a normal night of sleep. At 5 am, performance in most psychometric tests showed significant impairment. As expected, largest effect sizes were found on performance in the Psychomotor Vigilance Test (PVT). Large effects sizes were also found in the Divided Attention Test (DAT), the Attention Network Test (ANT), and the test for Useful Field of View (UFOV) at 5 and 11 am during sleep deprivation. Effects of sleep deprivation on SDLP correlated significantly with performance changes in the PVT and the DAT, but not with performance changes in the UFOV.

**Conclusion:**

From the psychometric tests used in this study, the PVT and DAT seem most promising for initial evaluation of drug impairment based on sensitivity and correlations with driving impairment. Further studies are needed to assess the sensitivity and validity of these psychometric tests after benchmark sedative drug use.

## Introduction

Medicinal and illicit drugs can have detrimental side effects, such as sedation and reduced alertness, which can cause driving impairment possibly leading to traffic accidents (e.g. [1], [2], [3], [4], [5]). Performance testing should be applied to provide meaningful precautions for users and prescribers regarding the impact of particular drugs on driving, either as part of the drug registration process (e.g. [6]) or for already marketed medicinal or illicit drugs (e.g.[7]). Methodological guidelines for experimental studies assessing the effects of drugs on driving indicate that relatively simple laboratory tests can be used as a first step in screening a drug’s impairing potential as they often provide the earliest evidence of impairment on driving performance [8], and that more sophisticated procedures (e.g. driving simulators, on-the-road testing) should be included in a later stage [9], [10], [11], [12]. The advantage of simple laboratory tests is that these tests are generally easy to administer, are cost-effective, and have a relative short duration [9], [11], [12], [13]. Many tests are being used to indicate whether drugs impair driving performance and to judge fitness to drive when drugs are being used. However, no consensus has been reached about which specific initial screening tools are best to be used [10], as the link between test outcomes and clinical relevance is often unclear.

Therefore, it is needed to establish a link between tests and effects of clinical relevance in order to compare results over separate studies. To provide this information, a requisite of a test is to be sufficiently sensitive to detect clinically relevant impairment [5], [10]. Drugs with known impairing effects can be used to induce clinically relevant levels of impairment, such as alcohol. Epidemiological studies have indicated an increase of traffic accidents with alcohol reaching blood alcohol concentrations of 0.5 g/L [14], [15]. However, when a psychometric test is not sensitive to the impairing effects of clinically relevant levels of alcohol, it does not necessarily mean it is not sensitive to more specific drug effects.

Another way to establish the clinically relevant performance impairment of a test is to assess its sensitivity to clinically relevant levels in drowsiness induced by one night of sleep deprivation [16], [17]. After alcohol, sleepiness is the most frequent cause of motor vehicle accidents [18], [19]. Being sleep deprived while driving is a serious problem and is a direct or contributing factor in road related accidents [20], [21], [22]. Many experimental studies have confirmed that driving related skills, such as vigilance and divided attention, deteriorates under conditions of sleep deprivation [23], [24], [25], [26], [27], [28], [29]. The present study includes sleep deprivation to induce clinically relevant levels of sedation to assess relevant impairment.

A number of psychometric tests are often used to assess possible driving impairment, but the choice of tests differs depending on the area of research or practice. The Psychomotor Vigilance Test (PVT) is often used in sleep research for assessing drowsiness resulting from disturbed or insufficient sleep [30], [31], [32]. The Critical Tracking Test (CTT), Divided Attention Test (DAT), the Digit Symbol Substitution Test (DSST) and the Determination Test (DT) as part of the Vienna Test System [33] are often used in psychopharmacological studies to assess drug induced effects in healthy volunteers or patients [8], [11], [34], [35], [36], [37], [38]. A Postural Balance Test (PBT) has been indicated as a feasible test to assess drowsiness at the roadside [39]. Furthermore, several of these tests (i.e. PVT, DAT, and PBT) are sensitive to the impairing effects of clinically relevant levels of alcohol reaching a Blood Alcohol Concentration (BAC) of 0.5 g/L [40].

In the field of ageing and dementia, The Concept Shifting Test (CST) or the equivalent Trail Making Test [41], [42] and a test of Useful Field of View (UFOV) [43] are used to assess driving impairment. The Attention Network Test (ANT) is used in the field of neuropsychology [44] as it measures the efficiency of multiple attention networks.

After initial screening, measures of driving with higher ecological validity are often used to assess drug induced impairment. The standardized highway driving test used in the Netherlands [1], [2], [13] is a sensitive and reliable measure to assess drug induced impairment. Standard Deviation of Lateral Position (SDLP) is the primary outcome of this test and has been proven to be sensitive to the effects of many sedative drugs, such as alcohol [45], [46], antidepressants [8], antihistamines [47], [48], the residual effects of hypnotics [49], [50], [51], and was sensitive to the effects of sleep deprivation [52]. Therefore, the highway driving test is used as a reference to indicate the magnitude of the effect of clinically relevant levels of impairment after one night of sleep deprivation. Laboratory tests showing comparable effect sizes could be useful as initial screening tools to assess drug induced driving impairment.

In addition, laboratory tests can be useful when they are able to predict actual driving impairment. Two reviews correlated drug-induced changes driving performance (SDLP) and performance in a number of psychometric tests [8], [53]. Ramaekers [8] calculated intrasubject correlations (n = 32) between changes in driving and psychomotor task performance across treatment conditions. Correlations were assessed in two separate studies showing mild and strong drug effects of antidepressant drugs (i.e. doxepin 75 mg and amitriptyline 75 mg, respectively). Tests of critical tracking, divided attention, choice reaction time, critical flicker fusion, memory, finger tapping, and vigilance were included. Results showed several significant correlations, but these were relatively modest. The highest correlations (r = 0.45) were found between SDLP and tracking performance as measured by the CTT and the DAT for strong drug effects. The strength of the associations depended on the severity of the drug effects; correlations diminished with milder drug effects.

Verster and Roth [53] analysed data from three studies (n = 96) showing varying effects of hypnotics, antihistamines, analgesics, and alcohol on actual driving and performance in tests of tracking, divided attention, memory and digit symbol substitution. Similar to the findings of Ramaekers [8], highest correlations with changes in SDLP were found for tracking in a continuous tracking test and a divided attention test (overall r = 0.47). Nevertheless, regression analysis showed that the combination of all performance parameters explained only 33% of the variance found in driving. Notably, the strength of the associations depended again on the severity of the impairing effects.

The main objective of the present study was to determine the ability of nine psychometric tests to detect clinically relevant impairing effects of drowsiness as induced by one night of sleep deprivation. More specifically, the sensitivity of these laboratory tests was assessed during a single night of sleep deprivation at 1:00 am, 5:00 am, and 11:00 am, i.e. after 16, 20 and 26 hours of wakefulness and compared with performance after a night of normal sleep. A secondary objective was to determine and compare the magnitude of the sedative effects on these tests during and after a night of sleep deprivation for future reference in clinical trials. We hypothesized that all these tests would be sensitive to the impairing effects of one night of sleep deprivation, but that the magnitude of the impairing effect would differ between tests. The magnitude of the effects in the laboratory tests was compared with the magnitude of effect on SDLP in the highway driving test. We expected a large effect of sleep deprivation on SDLP in the highway driving test. A third objective was to determine the correlations between performance changes in these tests and those in driving performance to evaluate the validity of each test for predicting driving impairment (i.e. SDLP changes).

## Methods

### Participants

Twenty-four healthy volunteers (12 males, 12 females) aged between 23–45 years were recruited through advertisements at Maastricht University. Initial screening was based on a medical history questionnaire. Eligible participants were invited for a physical examination, which included urinalysis, tests for drugs of abuse (amphetamines, benzodiazepines, cannabis, cocaine, 3,4-methylenedioxymethamphetamine, and opiates), and a 12-lead electrocardiogram. For participation, the following inclusion criteria had to be met: possession of a valid driving license for three years or more, driving experience of at least 5000 km per year on average over the last three years and a body mass index (BMI) between 19 and 29 kg mˉ². Exclusion criteria included: shift work; history of a sleep disorder; extreme morning or evening type; any history of psychiatric or medical illness; history or current drug or alcohol abuse; current use of psycho-active medication; excessive caffeine use, defined as drinking six or more cups of coffee per day.

The mean (± SD) age of the participants was 26.9 (± 3.4) years. The study was conducted in accordance with the code of ethics on human experimentation established by the declaration of Helsinki (1964) and amended in Seoul (2008). All participants were informed of the study’s goal, procedures, and potential hazards in writing, and they indicated their informed consent in writing. The Medical Ethics Committees of Maastricht University approved the study. Participants received a financial compensation for their participation in the study.

### Design

The study was conducted according to a 2-period cross-over design to compare performance after one night of sleep deprivation with performance after a normal night of sleep. The highway driving test was conducted twice: one after normal sleep and once after one night of sleep deprivation (i.e. 24h of wakefulness) both starting at 9:00 am. The psychometric tests were conducted on 4 occasions: once after normal sleep (at 11:00 am) and three times during a single night of sleep deprivation (at 1:00 am, 5:00 am, and 11:00 am, i.e. after 16h, 20h, and 26h of wakefulness). Both conditions were separated by an interval of at least one week, and the order was balanced over participants. To reduce order effects between the tests, the test battery was divided in two parts and these parts were balanced over participants.

### Procedure

Participants were individually trained to perform the behavioral tests prior to the first test day in two separate sessions. Participants agreed not to use any drugs of abuse or oral medication (except oral contraceptives and paracetamol) during the study. During participation in the study, alcohol intake was not allowed from 24 hours prior to each test day until discharge. On testing days, caffeine intake and smoking was not allowed until discharge. In both conditions, participants wore wrist actimeters and filled in sleep diaries to evaluate the rest/activity rhythm one week before testing.

**Fig 1:**
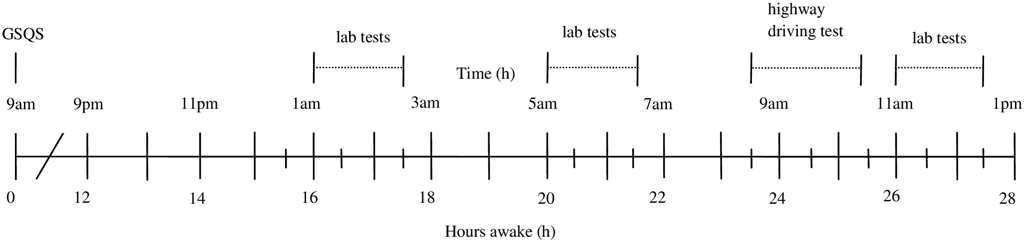
Timeline of the sleep deprivation condition. Abbreviation: GSQS = Groningen Sleep Quality Scale.

In the sleep deprivation condition, participants were called at 9:00 am to wake them up and to verify a night of good sleep assessed by the Groningen Sleep Quality Scale (GSQS, [54]). Participants arrived at the site at 9:00 pm in order to accompany them in complying with the procedures. They yielded urine and breathe samples to confirm their compliance with prohibitions against use of drugs and to verify a BAC of 0.0 g/L. At 1:00 am and 5:00 am, the participants performed the first and second session of the laboratory tests, comprising the Critical Tracking Test, Divided Attention Test, Psychomotor Vigilance Test, Digit Symbol Substitution Test, Attention Network Test, Concept Shifting Test, Postural Balance Test, Determination Test, and the test of Useful Field of View. At 8:00 am, a standardized breakfast was served. Participants were transported to the highway at 8:30 am. The highway driving test was conducted from 9:00 am to 10:00 am. Upon completion of the driving test the participants returned to the testing facilities for a third test session, starting at 11:00 am. See Fig. 1 for a timeline of testing during a single night of sleep deprivation.

After a night of normal sleep, participants arrived at the site at 07:45 am. The GSQS was administered and participants yielded urine and breathe samples to confirm their compliance with the protocol. The highway driving test was conducted from 9:00 am to 10:00 am. The participants returned to the testing facilities for one test session comprising the laboratory tests described above.

Both testing days ended at 13:00 pm. After normal sleep, participants were dismissed; after sleep deprivation, participants were driven home.

### Assessment

**Highway Driving Test**. In the standardized highway driving test [2, 13] the participant operates a specially instrumented vehicle over a 100-km (61-mile) primary highway circuit, accompanied by a licensed driving instructor having access to dual controls. The task of the participant is to maintain a constant speed of 95 km/h (58 m/h) and a steady lateral position between the delineated boundaries of the right traffic lane. The vehicle speed and lateral position are recorded continuously. These signals are digitized at a rate of 4 Hz and edited off-line to remove data recorded during overtaking manoeuvres or disturbances caused by roadway or traffic situations. The remaining data are then used to calculate mean values and standard deviation of lateral position and speed. The primary outcome variable is Standard Deviation of Lateral Position (SDLP, in cm) which is a measure of road tracking error, or ‘weaving’. The secondary outcome variable is the Standard Deviation of Speed (SDSP), which is an index of the ability to maintain a constant speed.

**Psychomotor Vigilance Task**. The Psychomotor Vigilance Task (PVT) is based on a simple visual reaction time test [55]. Mean reaction time (RT) in ms, inverse reaction time (1/RT), and lapses (i.e. RT > 500 ms) were calculated. 1/RT emphasizes slowing in the optimum and intermediate response domain and it substantially decreases the contribution of long lapses. For calculation mean 1/RT each RT (ms) was divided by 1,000 and then reciprocally transformed [56].

**Critical Tracking Task**. The Critical Tracking Task (CTT) measures the ability to control an unstable error signal in a first-order compensatory tracking task [57]. This test is designed to measure psychomotor coordination. Participants are instructed to keep an unstable cursor in the middle of a horizontal plane by counteracting or reverse its movements with the aid of a joystick. The frequency of cursor deviations at which the participant loses control is the critical frequency or lambda (λ_c_), in rad sˉ1. The CTT includes five trials to obtain a reliable mean performance score. The highest and lowest scores are removed, as extreme high or low scores could cause high skewness of the mean performance. The final score is the average of the three remaining scores.

**Divided Attention Task**. The Divided Attention Task (DAT) measures the ability to divide attention between two simultaneously performed tasks [36]. In the primary task, the participant performs the same tracking task described above, yet at a constant level of difficulty set at 50% of his or her maximum capacity, as measured by the individual’s best mean lambda score in the CTT at the end of training. In the secondary task, the participant monitors 24 peripheral displays in which single digits change asynchronously at 5-s intervals. Participants are instructed to remove their foot from a pedal as rapidly as possible whenever the digit “2” appears. This signal occurs twice at every location, in random order, at intervals of 5–25 seconds. The primary dependent measures in each subtask are tracking error (in mm) and average reaction time to targets (in ms). Secondary control measures are control losses in the tracking task and number of hits in the target detection task.

**Digit Symbol Substitution Test**. The Digit Symbol Substitution Test (DSST) measures many different psychomotor and cognitive functions at the same time [58]. A computerized version [59] of the original paper-and-pencil test taken from the Wechsler Adult Intelligence Scale is used (e.g. [60]). The participant is required to match each digit with a symbol from the encoding list as rapidly as possible by using a touch screen. The number of digits encoded correctly within 3 min is the performance measure.

**Attention Network Test**. The Attention Network Test (ANT) provides measures of three functions of attention within a single task [61]. Participants are instructed to keep their eyes fixed on a fixation cross throughout the test. Then, at some variable interval (ranging from 400 to 1600 ms) a cue is presented for 100 ms. After the offset of the cue, a target display appears, and remains on until response (i.e., a key-press indicating the direction of the target arrow), or for 1700 ms if no response is made. Dependent variables are total reaction time, alerting (i.e. difference between reaction time in no cue condition and double cue condition), orienting (difference between reaction time in center cue condition and spatial cue condition) and conflict (difference between reaction time with incongruent flankers and congruent flankers). The test duration is approximately 20 minutes. For a full description of the task, see the article of Fan and colleagues [61].

**Concept Shifting Task**. The computerized Concept Shifting Task (CST) is used to measure processing speed and cognitive flexibility [62]. It consists of three subtasks (A, B, and C). On each display 16 small circles are grouped into one larger circle. In the smaller circles the test items (numbers [A], letters [B] or both [C]) appear in a fixed random order. In part A, participants are asked to cross out numbers (1–16) in the right order as quickly and accurately as possible, using a touch screen. In part B, the circles contain letters (A–P) that have to be crossed out in alphabetical order. In part C, the both numbers and letters are displayed, and the participant is requested to alternate between numbers and letters. The time needed to complete each part is scored (CST-A, CST-B, CST-C in s, respectively). An interference score (CST_i_) was obtained by the following formula: (CST-C − ½ * (CST-A + CST-B)) / (½ * (CST-A + CST-B)) * 100.

**Postural Balance Test**. The Postural Balance Test (PBT) is measured by using the AMTI AccuSway System for Balance and Postural Sway Measurement (Advanced Mechanical Technology, Inc., Watertown, MA) force platform [63], [64]. Postural sway is assessed by measuring the length of the path of the centre-of-pressure (COP), and the area of the 95% confidence ellipse enclosing the COP (A95), which is the primary outcome measure in cm^2^. The test is conducted in two trials of both 60 seconds: one trial with the participants’ eyes open and one trial with eyes closed, both with feet apart at hip’s width.

**Determination Test**. The Determination Test (DT) [33] is used to measure resilience of attention and reaction speed under conditions of sensory stress. The task of the participant is to identify various stimuli and to react to them by pressing the respective corresponding response buttons, using the response panel of the Vienna Test System. The test is administered as a computerized adaptive test whereby the presentation time of the stimuli adjusts itself to the reaction speed of the participant. However, unlike classic computerized adaptive tests, this test form presents the stimuli a little faster than would be optimal given the participants’ reaction speed, thus resulting in a condition of sensory stress. Median reaction time and correct responses were used to assess the performance of the participants. Total duration of the test is approximately 4 min.

**Useful Field of View test**. The test of Useful Field of View (UFOV) is a computer-based test measuring detection time for three subtests (visual processing speed, divided attention, and selective attention) which involve attentional tasks of increasing difficulty [65]. A total detection time was computed by summing the threshold scores for the 3 subtests. Total duration of the test is approximately 7 min. For a full description of the task, see the article of Edwards and colleagues [65].

**Subjective rating scales**. The driving instructors rated each participant’s driving quality and sedation at the conclusion of the highway driving test, using two 100-mm visual analogue scales. Participants rated their subjective feeling of sleepiness and driving quality prior to, halfway, and at the conclusion of the Highway Driving Test using the Karolinska Sleepiness Scale (KSS, [66]) and a modified version of the Driving Quality Scale (DQS, [67]). The KSS is a nine-point rating scale ranging from extremely alert (1) to very sleepy, great effort to keep alert, fighting sleep (9). The modified DQS is a 10 point rating scale ranging from extremely poor (1) to extremely good (10).

### Statistical analyses

Sample size calculation was based on detecting a minimally relevant difference with an effect size of 0.25 between performance after a normal night of sleep and at 5:00 am, i.e. after 20 hours of wakefulness during a night of sleep deprivation. Given a test-retest reliability of parameters at the Psychomotor Vigilance Test of at least *r* = 0.80 [68], a group of 24 participants should permit detection of a mean change in reaction time, with a power of at least 95% and an α of .05.

For the highway driving test, each variable was analyzed using General Linear Model (GLM) repeated measures with wakefulness (two levels) as within subject factor. For the laboratory tests, each variable was analyzed using GLM repeated measures with wakefulness (four levels) as within subject factor. Three separate sleep deprivation-normal sleep contrasts were conducted when an overall effect of sleep deprivation was found.

Change scores for each of the dependent variables were transformed to z-scores, which were calculated across the pooled changes in the single night of sleep deprivation on three occasions relative to performance on a separate day after one night of normal sleep. This allows for easy comparison across each of the various performance tests [69]. To determine the magnitude of the simple effects at various times during the night of sleep deprivation, Dunlap et al.’s [70] effect size (ES) statistics (i.e. t_c_[2(1-r)/n]^1/2^) were calculated. This statistic is used to calculate effect sizes in repeated measure designs. Effect sizes between 0 and 0.19 are considered small, between 0.20–0.69 are considered moderate and 0.70 or higher are considered large [71].

To determine the correlations between changes in psychometric test performance and driving, Pearson’s correlations were calculated between changes scores of SDLP and psychometric performance immediately before and after driving, i.e. at 5 am and at 11 am. Only significant correlations are reported. All statistical analyses were done by using the Statistical Package for the Social Sciences for Windows (version 21; SPSS Inc, Chicago, IL, USA).

## Results

### Missing data

One participant withdrew from further testing at 5:00 am due to excessive sleepiness. His data were excluded for statistical analyses. Due to technical problems, no data were collected for one participant during the DT and for another during the DAT on a single occasion. Only participants with complete data sets were entered in the analysis of the respective performance parameters. Outliers, defined as a difference by more than two times the standard deviation from the mean, were removed.

### Effects of sleep deprivation on highway driving test

Table 1 summarizes the mean (SE) scores obtained in the highway driving test. Seven driving tests were terminated before scheduled completion, all in the sleep deprivation condition. One test was terminated by the driving instructor because he judged the participant to be too drowsy to continue safely. Six tests were terminated by participants because they felt too drowsy to continue safely. All premature terminations occurred between 30 and 60 minutes of the driving test. Of these driving tests, SDLP scores were calculated from the data collected until termination of the test.

**Table 1:** Mean (SE) and effects of one night of sleep deprivation in the on-the-road highway driving test.

	Time of day		Overall effect	
	9:00 am	9:00 am (+24 h)	F	Dunlap’s ES
SDLP	15.11 (0.6)	18.23 (0.7)	30.29***	0.97
SDSP	2.09 (0.08)	2.50 (0.14)	8.56**	0.73
Mean lateral position	95.70 (2.6)	97.15 (2.4)	0.76	0.12
Mean speed	93.37 (0.4)	92.69 (0.6)	2.34	0.27
Subjective sleepiness (KSS 1–9)				
Before driving	2.83 (0.2)	5.78 (0.4)	49.72***	2.17
At turning point	2.65 (0.3)	6.74 (0.3)	108.60***	2.89
After driving	3.28 (0.3)	6.94 (0.4)	82.28***	1.95
Subjective driving quality (1–10)				
First part	7.48 (0.2)	5.61 (0.4)	19.52***	1.32
Second part	7.44 (0.2)	5.72 (0.4)	13.83**	1.19
Instructor rating (VAS mm)				
Sedation	13.65 (2.1)	56.35 (6.0)	59.65***	1.79
Driving quality	25.96 (2.5)	41.00 (3.3)^a^	12.15**	1.06

Abbreviations: ES = Effect Size; SDLP = Standard Deviation of Lateral Position, SDSP = Standard Deviation of Speed, KSS = Karolinska Sleepiness Scale, VAS = Visual Analogue Scale.

*p <. 05,

**p <. 01,

***p <. 001

^a^ Increase of mm indicate worse subjective driving quality.

Analysis showed that mean SDLP was significantly increased by 3.1 cm after sleep deprivation compared to driving after a night of normal sleep (F_1, 22_ = 30.29, *p* <. 001). This increase corresponds to an effect size of 0.97. SDSP was significantly increased after one night of sleep deprivation as compared with driving after a normal night of sleep (F_1, 22_ = 8.56, *p* <. 01). This increase corresponds to an effect size of 0.73.

**Subjective rating scales**. In the sleep deprivation condition, participants felt significantly more sleepy than normal, as measured with the KSS before (F_1, 22_ = 49.72, *p* <. 001) halfway (F_1, 22_ = 108.60, *p* <. 001), and at the end (F_1, 22_ = 82.28, *p* <. 001) of the driving test. At the same time, participants judged their driving quality to be worse than normal during the first and second half of the driving test (F_1, 22_ = 19.52, *p* <. 001; F_1, 22_ = 13.83, *p* <. 001, respectively). In line with this, instructors judged subjects to appear more sedated (F_1, 22_ = 59.65, *p* <. 001) and drive worse (F_1, 22_ = 12.15, *p* <. 01) after one night of sleep deprivation compared with driving after a normal night of sleep.

### Laboratory tests

Table 2 presents a summary of the means and standard errors of the means (SE) of all performance scores, the results of the analyses of variance, and the simple contrasts.

**Table 2:** Mean (SE), Overall Effects of Sleep Deprivation and Contrast Analyses of Laboratory Tests.

	Normal night of sleep	Sleep deprivation			Overall effect		Simple contrasts		
	11:00 am	01:00 am	05:00 am	11:00 am			11:00 am versus 01:00 am	11:00 am versus 05:00 am	11:00 am versus 11:00 am
Test	Mean (SE)				F	p	p	p	p
Psychomotor Vigilance Test									
Inverse reaction time	3.96 (0.09)	4.02 (0.07)	3.57 (0.14)	3.44 (0.10)	22.19	<0.001	0.22	<0.001	<0.001
Mean reaction time (ms)	258 (4.9)	265 (6.6)	308 (17.2)	328.5 (13.5)	9.20	<0.01	0.09	<0.01	<0.001
Lapses	0.83 (0.30)	1.13 (0.28)	6.30 (2.06)	8.39 (1.68)	6.37	<0.01	0.44	0.02	<0.001
Critical Tracking Test									
Mean lambda (rad/s)	3.73 (0.12)	3.82 (0.11)	3.65 (0.15)	3.52 (0.16)	2.98	0.06	0.08	0.41	0.06
Divided Attention Test									
z-AE+z-lg10(cl)	-0.65 (0.29)	-0.95 (0.29)	0.31 (0.41)	1.27 (0.38)	18.54	<0.001	0.22	<0.01	<0.001
z-RT+ z-lg10(mi)	-0.48 (0.36)	-0.46 (0.33)	0.20 (0.50)	0.75 (0.44)	6.47	<0.01	0.92	0.05	<0.01
Attention Network Test									
Overall reaction time (ms)	492 (12)	492 (13)	552 (22)	586 (20)	11.73	<0.001	0.99	<0.01	<0.001
Alerting effect (ms)	53 (6)	55 (5)	59 (10)	69 (7)	1.52	0.24	0.69	0.48	0.06
Orienting effect (ms)	49 (4)	51 (4)	57(5)	32 (5)	2.30	0.11	0.22	0.72	0.27
Conflict effect (ms)	103 (6)	96 (6)	115 (9)	130 (10)	6.23	<0.01	0.10	0.17	0.03
Digit Symbol Substitution Test									
Correct responses	105.6 (3.0)	101.4 (3.1)	96.4 (3.3)	98.4 (3.6)	3.63	0.03	0.03	0.021	<0.01
Concept Shifting Test									
Reaction time CST-A (s)	18.6 (0.9)	19.1 (0.9)	19.0 (0.6)	19.0 (0.6)	0.65	0.59	0.59	0.20	0.42
Reaction time CST-B (s)	21.1 (0.9)	21.8 (0.9)	22.1 (0.7)	21.6 (0.7)	1.43	0.27	0.27	0.10	0.06
Reaction time CST-C (s)	24.9 (1.2)	25.7 (1.4)	28.3 (1.2)	24.9 (1.0)	3.22	0.05	0.05	0.36	<0.01
Interference (CST_i_)	25.7 (3.2)	24.8 (3.6)	38.2 (5.1)	22.8 (2.8)	2.04	0.14	0.79	0.04	0.53
Determination Test									
Correct responses	296.4 (9.0)	291.1 (9.1)	278.0 (12.3)	279.0 (12.8)	2.36	0.10	0.28	0.06	0.02
Median reaction time (ms)	646 (14)	665(14)	681 (18)	681 (20)	3.89	0.03	<0.01	<0.01	0.02
Useful Field of View Test									
Total detection time (ms)	80.6 (6.4)	86.3 (8.2)	140.0 (19.8)	131.4 (17.4)	4.26	0.02	0.38	<0.01	<0.01
Postural Balance Test									
Eyes open—ln-area 95 (cm^2^)	0.42 (0.16)	0.43 (0.16)	0.84 (0.13)	0.61 (0.14)	7.34	<0.01	0.91	<0.01	0.15
Eyes closed—ln-area 95 (cm^2^)	0.61 (0.1)	0.60 (0.1)	1.0 (0.2)	0.90 (0.2)	3.82	0.03	0.97	<0.01	0.04

Abbreviations: z-AE = z-score of average tracking error; z-lg10(cl) = z-score of log transformed total number of control losses; z-RT = z-score of reaction time; z-log10(mi) = z-score of log transformed total number of misses; ln = natural log.

**Fig 2:**
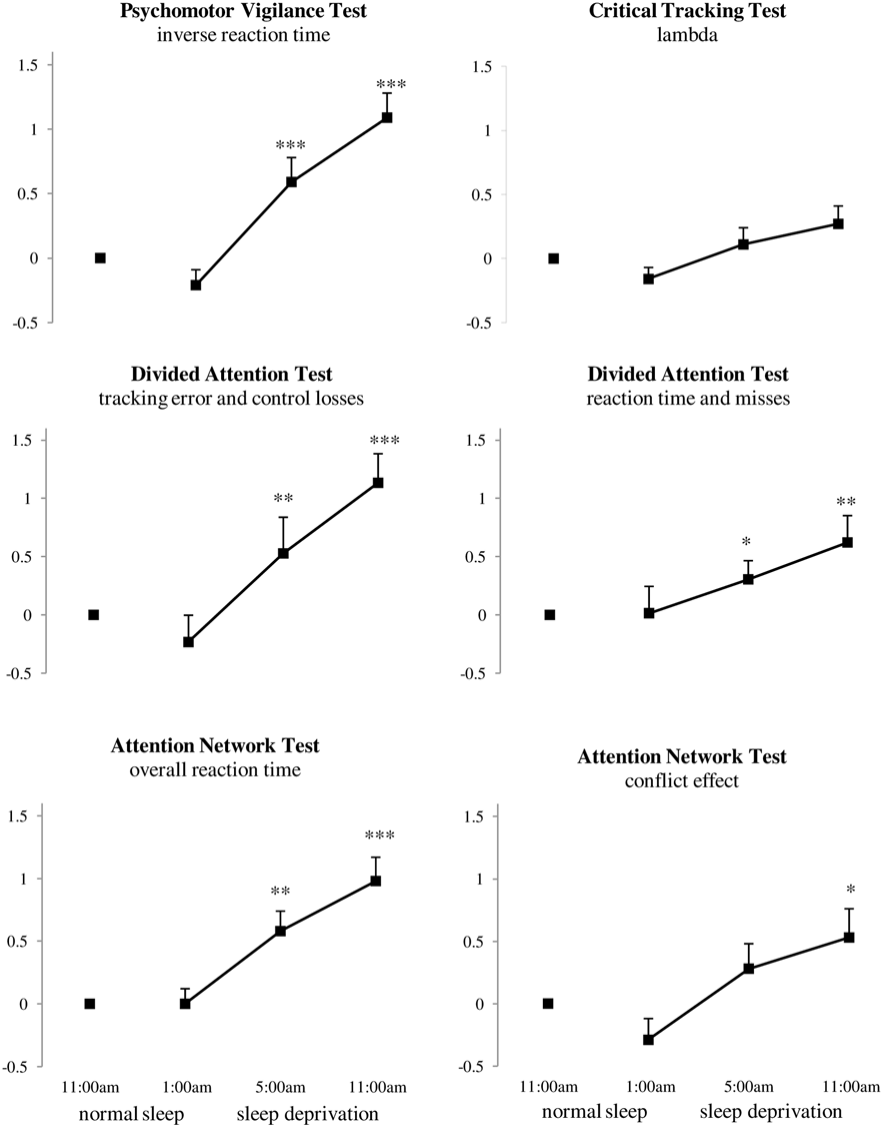
Mean baseline normalized performance at 1:00am (16 hours awake), 5:00am (20 hours awake) and 11:00 am (26 hours awake) compared with performance after a normal night of sleep (at 11:00 am) across dependent variables of the Psychomotor Vigilance Test, Critical Tracking Test, Divided Attention Test, and Attention Network Test. *p < 0.05, **p < 0.01, ***p< 0.001. Error bars indicate the standard error of the mean.

**Psychomotor Vigilance Test**. Mean reaction time (F_3, 18_ = 9.20, *p* <. 01) and lapses (F_3, 18_ = 6.37, *p* <. 01) in the PVT were significantly different between the conditions of sleep deprivation. Contrast analysis indicated an increase of mean reaction time at 5:00 am (F_1, 20_ = 10.95, *p* <. 01) and 11:00 am (F_1, 20_ = 30.30, *p* <. 001) during sleep deprivation compared with mean reaction time after a night of normal sleep. Lapses increased at 5:00 am (F_1, 22_ = 6.95, *p* <. 05) and 11:00 am (F_1, 22_ = 20.66, *p* <. 001) during sleep deprivation compared with lapses after a normal night of sleep. To decrease the contribution of long lapses, inverse reaction times (1/RT) were calculated. A main effect of sleep deprivation was found at 1/RT (F_3, 20_ = 22.19, *p* <. 001). Contrast analysis indicated a decrease of 1/RT at 5:00 am (F_1, 22_ = 24.22, *p* <. 001) and 11:00 am (F_1, 22_ = 44.26, *p* <. 001) during sleep deprivation compared with 1/RT after a normal night of sleep (see Fig. 2).

**Critical Tracking Test**. A trend towards a main effect of sleep deprivation was found on mean tracking performance in the CTT (F_3, 20_ = 2.98, *p* = .06). Contrast analysis revealed a trend towards an increase of mean tracking performance at 5:00 am (F_1, 22_ = 3.29, *p* = .08) and a trend towards a decrease of tracking performance at 11:00 am (F_1, 22_ = 4.00, *p* = .06) during sleep deprivation compared with performance after a normal night of sleep (see Fig. 2).

**Divided Attention Test**. Two participants were not able to perform the DAT (i.e. more than 100 control losses) at 11:00 am during sleep deprivation; therefore these participants were not considered for the analysis. The secondary control measures, control losses (F_3, 18_ = 4.85, *p* = .01) and misses (F_3, 18_ = 4.81, *p* = .01), were significant different between conditions of sleep deprivation. These variables are therefore taken into account for measuring primary and secondary task performance.

The distributions of control losses and misses were highly skewed. Therefore, transformations were applied to their logarithmic scores (log 10) before transformation to z-scores. Log 10 was applied to deal with zero values by using the formula NEWX = LG10 (X + 1). ANOVA of tracking performance (i.e. the sum scores of the z-scores of the average tracking error and log10 of the total number of control losses) revealed a significant main effect (F_3, 17_ = 18.54, *p* <. 001) of sleep deprivation. Contrast analyses indicated that tracking performance was significantly decreased at 5:00 am (F_1, 19_ = 8.76, *p* <. 01) and 11:00 am (F_1, 19_ = 53.29, *p* <. 001) during sleep deprivation compared with tracking after a normal night of sleep (see Fig. 2).

A significant main effect in target detection performance (i.e. a sum of the z-scores of reaction time and log10 of total number of misses) was found (F_3, 17_ = 6.47, *p* <. 01). Contrast analyses indicated that target detection performance was significant decreased at 5:00 am (F_1, 19_ = 4.48, *p* <. 05) and 11:00 am (F_1, 19_ = 14.50, *p* = .001) during sleep deprivation compared with target detection performance after a normal night of sleep (see Fig. 2).

**Fig 3:**
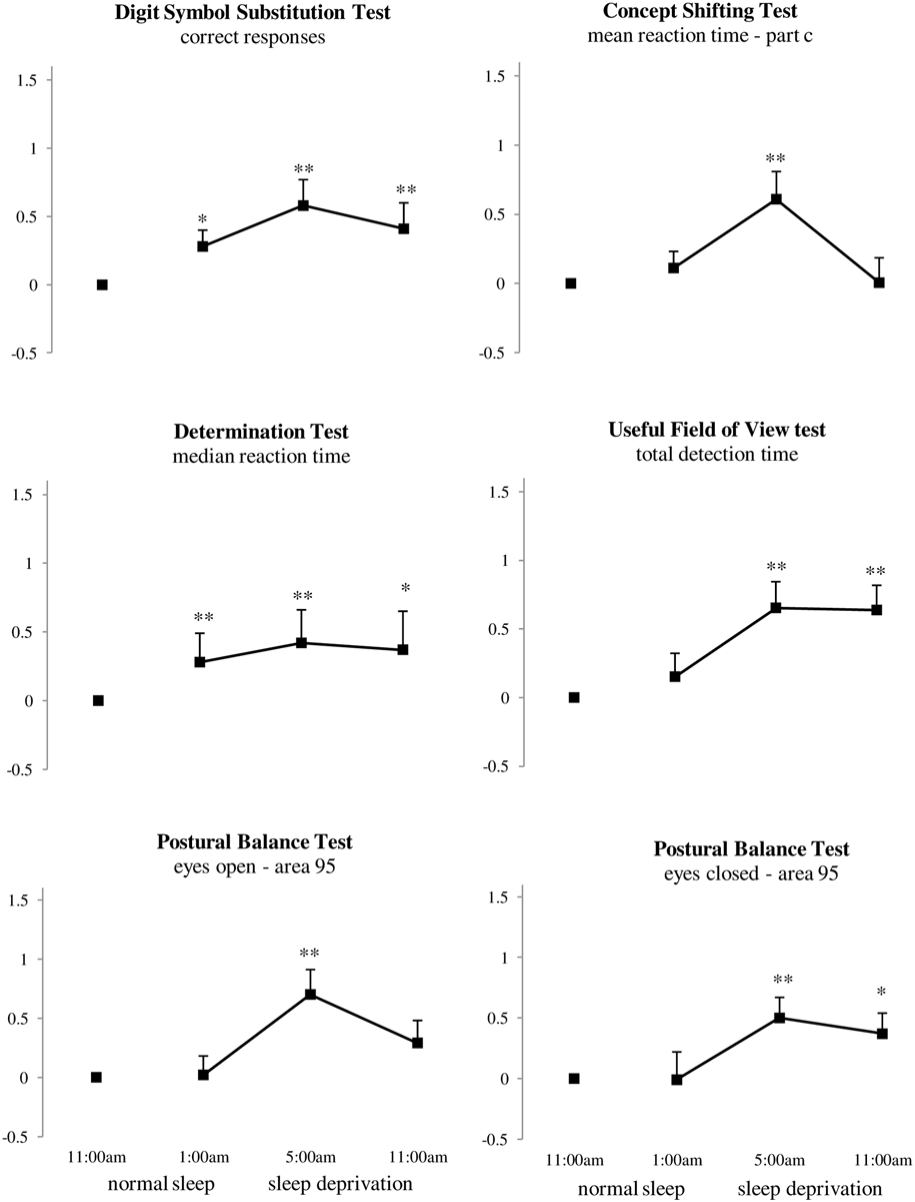
Mean baseline normalized performance at 1:00 am (16 hours awake), 5:00 am (20 hours awake) and 11:00 am (26 hours awake) compared with performance after a normal night of sleep (at 11:00 am) across dependent variables of the Digit Symbol Substitution Test, Concept Shifting Test, Determination Test, Useful Field of View Test, and Postural Balance Test. *p < 0.05, **p < 0.01, ***p <0.001. Error bars indicate the standard error of the mean.

**Attention Network Test**. A main effect of sleep deprivation was found on total reaction time in the ANT (F_3, 20_ = 11.73, *p* <. 001). Total reaction time increased at 5:00 am (F_1, 22_ = 13.79, *p* = .001) and 11:00 am (F_1, 22_ = 27.22, *p* <. 001) during sleep deprivation compared with total reaction time after a normal night of sleep (see Fig. 3). The conflict effect was different between conditions of sleep deprivation (F_3, 20_ = 6.23, *p* <. 01). The conflict effect increased at 11:00 am (F_1, 22_ = 5.08, *p* <. 05) during sleep deprivation compared with the conflict effect after a night of normal sleep. No main effects of sleep deprivation were found for the alerting and orienting effect (see Fig. 2).

**Digit Symbol Substitution Test**. The amount of correct responses in the DSST was significantly different between the conditions of sleep deprivation (F_3, 20_ = 3.63, *p* <.05). Participants’ correct responses decreased significantly at 1:00 am (F_1, 22_ = 6.17, *p* <. 05), 5:00 am (F_1, 22_ = 11.13, *p* <. 01), and 11:00 am (F_1, 22_ = 9.32, *p* <. 01) during a night of sleep deprivation compared with correct responses after a normal night of sleep (see Fig. 3).

**Concept Shifting Test**. A main effect of sleep deprivation was found on subtest C in the CST (F_3, 20_ = 3.22, *p* <. 05). Reaction time of part C increased at 5:00 am (F_1, 22_ = 8.95, *p* <. 01) during sleep deprivation compared with reaction time of part C after a normal night of sleep (see Fig. 3). No main effects of sleep deprivation were found at the subtests A and B and at the interference score (CST_i_).

**Determination Test**. Median reaction time at the DT was significantly different between the conditions of sleep deprivation (F_3, 19_ = 3.89, *p* <. 05). Contrast analysis revealed that median reaction time increased at 1:00 am (F_1, 21_ = 9.59, *p* <. 01), 5:00 am (F_1, 21_ = 8.30, *p* <. 01), and 11:00 am (F_1, 19_ = 7.03, *p* <. 05) during sleep deprivation compared with median reaction time after a night of normal sleep (see Fig. 3). No main effects were found on the other variables (i.e. correct responses, misses, and mistakes).

**Useful Field of View test**. A main effect of sleep deprivation was found on the third subtest (i.e. selective attention) of the UFOV (F_3, 18_ = 5.05, *p* = .01). Detection time at the selective attention test increased at 5:00 am (F_1, 20_ = 12.47, *p* <. 01) and 11:00 am (F_1, 20_ = 11.93, *p* <. 01) during sleep deprivation compared with detection time at the selective attention test after a night of normal sleep. No main effects were found on subtest one (i.e. visual processing speed) and two (i.e. divided attention) of the UFOV, although a trend was found toward a main effect of sleep deprivation on the divided attention subtest (F_3, 17_ = 3.14, *p* = .052).

A main effect of sleep deprivation was found on the total score of the UFOV (F_3, 18_ = 4.26, *p* <. 05). Contrast analysis revealed that total detection time increased at 5:00 am (F_1, 20_ = 11.62, *p* <. 01), and 11:00 am (F_1, 20_ = 12.13, *p* <. 01) during sleep deprivation compared with total detection time after a night of normal sleep (see Fig. 3).

**Postural Balance Test**. As data of the area of the 95% confidence ellipse enclosing the COP (A95) in the PBT was not normally distributed, data was log transformed (e.g. [72]). Main effects of sleep deprivation were found on A95 with both eyes open (F_3, 19_ = 7.34, *p* <. 01) and eyes closed (F_3, 19_ = 3.82, *p* <. 05). Contrast analysis revealed this effect was due to performance at 5:00 am (F_1, 21_ = 11.67, *p* <. 01) compared with A95 with eyes open after a night of normal sleep (see Fig. 3). With eyes closed, contrast analysis revealed this effect was due to performance at 5:00 am (F_1, 21_ = 8.53, *p* <. 01) and 11:00 am (F_1, 21_ = 4.64, *p* <. 05) during sleep deprivation (see Fig. 3).

### Comparison of performance measures

A summary of the mean difference with 95% confidence intervals, mean baseline-normalized z-scores, and Dunlap’s effect sizes (ES) is shown in Table 3. Effect sizes and z-scores indicate that tasks and parameters differ in sensitivity to the effects of one night of sleep deprivation. At 11:00 am after a night of sleep deprivation, largest effect sizes were found in the DAT on primary task performance and false alarms in secondary task performance (ES = 1.26 and 0.86, respectively), inverse reaction time, mean reaction time and lapses in the PVT (ES = 1.13, 0.98, and 1.23, respectively), overall reaction time in the ANT (ES = 1.13) and total detection time in the UFOV (ES = 0.70). At 5:00 am during the night of sleep deprivation, all these tests showed smaller effect sizes (0.60 ≤ ES ≤ 0.88) compared with 11:00 after a night of sleep deprivation. At 5:00 am, effect sizes were moderate (0.56 ≤ ES ≤ 0.62) on the test parameters of the CST and PBT, but smaller (0.00 ≤ ES ≤ 0.41) at 11:00 am during sleep deprivation.

**Table 3:** Mean difference scores with 95% confidence intervals with performance after normal night of sleep, mean baseline*-normalized z-scores, and effect sizes (Dunlap’s) of the performance tests.

	01:00 am (16h awake)		05:00 am (20h awake)		11:00 am (26h awake)		1:00 am	5:00 am	11:00 am	11:00 am versus 01:00 am	11:00 am versus 05:00 am	11:00 am versus 11:00 am
Test		95% CI		95% CI		95% CI	z-scores			ES	ES	ES
Psychomotor Vigilance Test												
Inverse reaction time	+0.06	-0.04 to 0.17	-0.39	-0.55 to-0.22	-0.51	-0.67 to-0.35	-0.21	0.59	1.09	-0.15	0.60^+^	1.13^++^
Mean reaction time (ms)	+6	-1 to 15	+50	18 to 81	+70	44 to 97	0.23	0.47	0.69	0.21^+^	0.62^+^	0.98^++^
Lapses	+0.30	-0.49 to 1.10	+5.48	1.17 to 9.79	+7.57	4.11 to 11.02	0.22	0.68	1.26	0.22^+^	0.78^++^	1.23^++^
Critical Tracking Test												
Mean Lambda (rad/s)	+0.09	-0.01 to 0.19	-0.08	-0.27 to 0.12	-0.21	-0.42 to 0.01	-0.16	0.11	0.27	-0.16	0.11	0.29^+^
Divided Attention Test												
z-AE+z-lg10(cl)	-0.30	-0.80 to 0.20	+0.96	0.28 to 1.63	+1.92	1.37 to 2.47	-0.23	0.53	1.13	-0.22	0.62^+^	1.26^++^
z-RT+ z-lg10(mi)	+0.02	-0.45 to 0.49	+0.68	0.01 to 1.36	+1.23	0.55 to 1.90	0.01	0.31	0.62	0.01	0.32^+^	0.67^+^
Attention Network Test												
Overall reaction time (ms)	0	-16 to 17	+60	27 to 94	+94	57 to 132	0.00	0.58	0.98	0.00	0.63^+^	1.13^++^
Alerting Effect (ms)	+2	-7 to 10	+6	-12 to 24	+16	-1 to 33	0.07	0.14	0.48	0.06	0.16	0.51^+^
Orienting Effect (ms)	+2	-7 to 10	+8	-3 to 18	-2	-16 to 12	0.08	0.33	-0.07	0.08	0.34^+^	-0.08
Conflict Effect (ms)	-8	-17 to 2	+12	-5 to 29	+26	2 to 50	-0.29	0.28	0.53	-0.26	0.32^+^	0.63^+^
Digit Symbol Substitution Test												
Correct responses	-4.2	-7.7 to-0.69	-9.2	-14.9 to-3.5	-7.2	-12.0 to-2.3	0.28	0.58	0.41	0.28^+^	0.60^+^	0.43^+^
Concept Shifting Test												
Reaction time CST-A (s)	+0.5	-0.3 to 1.4	+0.5	-0.7 to 1.6	+0.4	-0.9 to 1.8	0.13	0.16	0.14	0.13	0.12	0.11
Reaction time CST-B (s)	+0.7	-0.1 to 1.6	+1.0	-0.1 to 2.1	+0.5	-0.8 to 1.7	0.17	0.29	0.13	0.16	0.23^+^	0.11
Reaction time CST-C (s)	+0.7	-0.9 to 2.4	+3.4	1.0 to 5.7	0.0	-1.8 to 1.8	0.11	0.61	0.01	0.11	0.59^+^	0.00
Interference (CST_i_)	-0.9	-8.0 to 6.2	+12.5	0.6 to 24.4	-2.9	-12.3 to 6.5	-0.05	0.53	-0.21	-0.06	0.62^+^	-0.20^+^
Determination Test												
Correct responses	+18	6 to 30	+35	10 to 60	+34	7 to 61	0.28	0.42	0.37	0.13	0.35^+^	0.29^+^
Median reaction time (ms)	-5.3	-15.2 to 4.6	-18.5	-37.8 to 0.9	-17.5	-32.1 to-2.8	0.12	0.32	0.29	0.28^+^	0.45^+^	0.39^+^
Useful Field of View Test												
Total detection time (ms)	+5.7	-7.7 to 19.1	+59.4	23 to 96	+50.8	20.4 to 81.3	0.15	0.65	0.64	0.17	0.75^++^	0.70^++^
Postural Balance Test												
Eyes open—ln-area 95 (cm^2^)	+0.01	-0.24 to 0.27	+0.42	0.17 to 0.68	+0.19	-0.07 to 0.45	0.16	0.38	0.06	0.02	0.62^+^	0.27^+^
Eyes closed—ln-area 95 (cm^2^)	-0.00	-0.26 to 0.25	+0.39	0.11 to 0.67	+0.30	0.01 to 0.58	-0.01	0.50	0.37	0.01	0.56^+^	0.41^+^

+ indicates moderate effect size;

++ indicates large effect sizes.

Abbreviations: RT = Reaction Time; z-AE = z-score of average tracking error; z-lg10(cl) = z-score of log transformed total number of control losses; z-RT = z-score of reaction time; z-log10(mi) = z-score of log transformed total number of misses; ln = natural log.

* baseline is performance after a night of normal sleep on a separate day.

At 1:00 am during the night of sleep deprivation, two test parameters, correct responses in the DSST and median reaction time in the DT, showed moderate effect sizes (both ES = 0.28). These parameters showed moderate effects at 5:00 am and 11:00 am during and after a night of sleep deprivation: 0.60 and 0.43 on correct responses in the DSST and 0.45 and 0.39 on median reaction time in the DT, respectively.

Smallest effect sizes, i.e. 0.11 and 0.29, were found on lambda in the CTT at 5:00 am and 11:00 am, respectively, during sleep deprivation, respectively.

### Correlations between laboratory tasks and highway driving test

**Fig 4:**
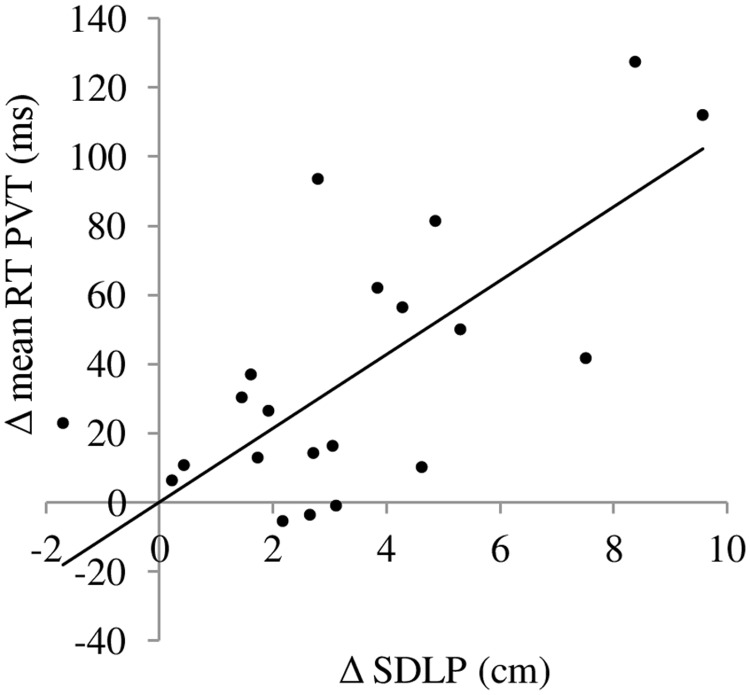
Correlations between changes (performance at 5:00 am—performance after normal night of sleep) in mean reaction time in the Psychomotor Vigilance Test and changes in SDLP.

Table 4 summarizes the significant correlations between changes in laboratory tasks and changes in the on-the-road highway driving test. Changes of mean reaction time in the PVT between 5:00 am and performance after a normal night of sleep correlated with changes in SDLP (r = 0.68, *p* <. 01) (see Fig. 4). SDLP changes significantly correlated with changes in lapses (r = 0.66, *p* <. 01) and inverse reaction time (r = -0.52, *p* <. 05) in the PVT between 5:00 am and performance after a normal night of sleep. Moderate correlations were found between changes of SDLP and changes of total mean reaction time in the ANT (r = 0.48, *p* <. 05), median reaction time in the DT (r = 0.47, *p* <. 05), and secondary task performance in the DAT (r = 0.47, *p* <. 05) between 5:00 am during sleep deprivation and performance after a normal night of sleep.

**Table 4:** Significant correlations between changes in laboratory test parameters and changes in on-the-road highway driving parameters between time points during a night of sleep deprivation and testing after a normal night of sleep.

Δ SDLP				
Test	Variable	Time of day during sleep deprivation	n^a^	Pearson’s r
Psychomotor Vigilance Test	Mean reaction time	5:00 am	21	0.68**
	Lapses	5:00 am	21	0.66**
	Inverse reaction time	5:00 am	21	-0.52*
Attention Network Test	Total mean reaction time	5:00 am	23	0.48*
Determination Test	Median reaction time	5:00 am	20	0.47*
Divided Attention Test	Mean reaction time and misses	5:00 am	20	0.47*
Δ SDSP				
Determination Test	Correct responses	5:00 am	20	-0.56**
	Median reaction time	5:00 am	20	0.55*
Digit Symbol Substitution Test	Correct responses	11:00 am	23	-0.42*

^a^ outliers, i.e. values larger than ± 2 SD from the mean, were removed.

*p <. 05,

**p <. 01.

Abbreviations: SDLP = Standard Deviation of Lateral Position, SDSP = Standard Deviation of Speed.

Moderate correlations were found between changes in SDSP and changes of correct responses (r = -0.56, *p* <. 01) and median reaction time (r = 0.55, *p* <. 05) in the DT between 5:00 am and performance after a normal night of sleep. Changes in SDSP correlated moderately with changes of correct responses in the DSST (r = -0.42, *p* <. 05) between 11:00 am during sleep deprivation and performance after a normal night of sleep.

## Discussion

The main objective of the present study was to determine the ability of nine psychometric tests to detect performance impairing effects of clinically relevant effects of drowsiness as induced by one night of sleep deprivation. After one night of sleep deprivation, performance at all psychometric tests except the CTT, was decreased. Specifically, performance in the DT and DSST was decreased at 1:00 am. At 5:00 am during a night of sleep deprivation, performance was impaired in the PVT, DAT, ANT, UFOV, DT, PBT, CST and DSST. At 11:00 am after a night of sleep deprivation, performance was impaired in all these tests, except the CST. The magnitude of effect was different among the tests at 5:00 am and 11:00 am during and after a night of sleep deprivation; largest effect sizes (i.e. ≥ 0.70) were found at the PVT, DAT, UFOV, and ANT.

In the highway driving test, a large impairing effect was found on SDLP after one night of sleep deprivation. SDLP increased with 3.1 cm compared with SDLP after a normal night of sleep, which is comparable to impairment at a BAC between 0.5 and 0.8 g/L [45] indicating the clinically relevant impairment after one night of sleep deprivation.

As expected, lapses and inverse reaction time in the PVT were among the most sensitive parameters to the impairing effects of being awake for 20 and 26 hours, which is in line with previous studies (e.g. [26], [30], [31], [32], [55]). The PVT was previously found to be sensitive to alcohol doses reaching clinically relevant BAC of 0.5 g/L [40] and lower [73]. The PVT is a promising screening tool to detect impairment, although it has not yet been shown to be sensitive to the impairing effect of medicinal drugs, such as zopiclone 7.5 mg [74]. Previous studies have consistently shown that the residual effects of zopiclone 7.5 mg impair driving performance, as indicated by an increase of SDLP comparable to impairment with a BAC of 0.5 g/L [49], [51]. Further research should examine the sensitivity of the PVT to benchmark sedative drugs to indicate its ability to detect specific drug effects.

The effects of sleep deprivation at the DAT are in line with previous studies [24], [27]. The DAT has previously been shown to be sensitive to the effects of many sedative drugs such as doses of alcohol, antidepressants, antihistamines, and the residual effects of hypnotics [37], [40], [46], [48], [60], [74]. The present results support the use of the DAT as screening tool for assessing clinically relevant driving impairment.

In the present study, performance at an UFOV test was impaired after sleep deprivation, which is in line with a previous study assessing these effects on performance in the UFOV while driving in a simulator [75]. The UFOV test seems a promising screening tool to assess drug induced impairment, although no significant effects were found in a previous study with alcohol reaching a clinically relevant blood alcohol level of 0.48 g/L [69]. Further research should therefore assess the sensitivity of the UFOV test to the specific effects of sedative drugs.

The ANT was also one of the most sensitive tests after sleep deprivation in the current study. The sensitivity of the ANT after sleep deprivation is in line with previous studies [76] [77]. Although total reaction time and the conflict effect were affected, the orienting and alerting effects were not increased after sleep deprivation. Thus, overall performance at the ANT is sensitive to the impairing effects of sedation, while specific effects at the networks of attention are less sensitive to indicate impairment. Furthermore, no effects of an alcohol dose reaching a clinically relevant level of 0.5 g/L were found in a previous study [40]. In addition, the ANT has a relative long duration (i.e. 20 min). Thus, other tests with a shorter duration measuring reaction time could be preferred to screen for relevant impairment.

The DSST, DT, and PBT were sensitive to the impairing effects of one night of sleep deprivation, but effect sizes were only modest. In these tests, performance slightly improved in the morning, as effect sizes were lower after 26 hours of wakefulness compared with effect sizes after 20 hours of wakefulness. This finding of a slight improvement in the morning was previously indicated in a cognitive psychomotor task [78]. This could be due to circadian effects, as assessing performance at 11:00 am is at a more favorable circadian phase compared to 5:00 am [79]. Therefore, the time of day should be considered when using these tests as screening tools to assess drug induced impairment.

A potential limitation of the use of laboratory tasks to assess driving impairment is their lack of validity for measuring driving and predicting accident risk [80]. The present study found relatively high correlations between performance changes in the PVT and changes in the highway driving test due to sleep deprivation. In contrast, analysis of drug induced impairment found only modest correlations [8], [53]. The high correlations found in the present study could be explained by the type of task and the type of manipulation. Regarding the type of task, both the PVT and the highway driving task require the ability to sustain attention over a longer period. In addition, both tests are known to be very sensitive to the effects of sleep deprivation [24], [52]. Correlations tend to increase with stronger impairing effects, as shown by drug studies [8], [53]. Drugs may have different or more subtle effects than a night of sleep deprivation. In addition, safe driving does not only depend on the ability to remain vigilant. Further studies should therefore be conducted to determine the ability of the PVT and other tasks to detect clinically relevant drug induced impairment.

To summarize, largest effects of sleep deprivation were found on performance in the PVT, DAT, ANT and UFOV. These effects were comparable to or larger than the effect on SDLP in the highway driving test. Furthermore, these tests were minimally influenced by circadian effects, as performance impairment was larger at 11:00 am compared with 5:00 am during sleep deprivation. In addition, performance changes in the PVT, DAT and ANT correlated significantly with changes in SDLP. Performance changes in the UFOV did not correlate with changes in driving and the duration of the ANT is considerably long compared with the other tests. The PVT and DAT seem therefore more preferable than the UFOV and the ANT.

According to the present study, effects of clinically relevant levels of drowsiness as induced by one night of sleep deprivation can be used as minimally relevant effects of impairment. One limitation of the implications of the present study is, however, that sleep deprivation, alcohol, and sedative drugs have qualitatively differences [81], [82]. Sedative drugs could have more specific effects on simple laboratory tests. Further studies with a double-blind, placebo-controlled, cross-over design examining the effects of benchmark sedative drugs are helpful to assess the sensitivity of laboratory tests to the potentially impairing effects and whether specific tests can predict drug induced driving impairment.

In conclusion, from the psychometric tests used in this study, the PVT and DAT seem most promising for initial evaluation of drug induced impairment based on sensitivity, correlations with driving impairment, and short duration. The effects of one night of sleep deprivation on these tests are similar to or larger than clinically relevant levels of alcohol [40]. Such decreases in arousal after one night of sleep deprivation are clinically relevant, as an increased crash risk has been indicated at night or in the early morning hours [20], [21], [22]. The suggested initial screening tools can be used as a first step to provide meaningful precautions for users and prescribers about the impact of drugs on driving.

## References

[1] O’Hanlon JF, Haak TW, Blaauw GJ, Riemersma JB (1982) Diazepam impairs lateral position control in highway driving. Science 217(4554): 79–81. 7089544 10.1126/science.7089544

[2] O’Hanlon JF (1984) Driving performance under the influence of drugs: rationale for, and application of, a new test. Br J Clin Pharmacol 18 Suppl 1: 121–129. 6525328 10.1111/j.1365-2125.1984.tb02590.x PMC1463355

[3] Seppala T, Linnoila M, Mattila M (1979) Drugs, alcohol and driving. Drugs 17(5): 389 37067 10.2165/00003495-197917050-00008

[4] Vingilis E, Macdonald S (2002) Review: Drugs and Traffic Collisions. Traffic Inj Prev 3(1): 1–11.

[5] Walsh JM, de Gier JJ, Christopherson AS, Verstraete AG (2004) Drugs and driving. Traffic Inj Prev 5(3): 241–253. 15276925 10.1080/15389580490465292

[6] O’Hanlon J (1986) Performance testing as part of drug registration In: Drugs and Driving. London: Taylor and Francis 402 p.

[7] Verstraete A, Knoche A, Jantos R, Skopp G (2011) Per se limits-Methods of defining cut off values for zero tolerance. Deliverable 142, DRUID (Driving under the Influence of Drugs, Alcohol and Medicines) 6th framework programme. Available at http://www.druid-project.eu/ Accessed 10 December 2013.

[8] Ramaekers JG (2003) Antidepressants and driver impairment: empirical evidence from a standard on-the-road test. The Journal of clinical psychiatry 64(1): 20–29. 12590619

[9] International Council on Alcohol Drugs and Traffic Safety (ICADTS) (1999) Guidelines on experimental studies undertaken to determine a medicinal drug’s effect on driving or skills related to driving. Bundes Anstalt fuer Strassenwesen (BASt), Cologne, p 1–13.

[10] Kay G, Logan B (2011) Drugged Driving Expert Panel Report: A Consensus Protocol for Assessing the Potential of Drugs to Impair Driving. Washington DC: National Highway Traffic Safety Administration 22553853

[11] Vermeeren A, De Gier JJ, O’Hanlon JF (1994) Methodological guidelines for experimental research on medicinal drugs affecting driving performance: an international expert survey. J Traffic Med, 22(4), 173–174.

[12] Walsh JM, Verstraete AG, Huestis MA, Mørland J (2008) Guidelines for research on drugged driving. Addiction 103(8): 1258–1268. 10.1111/j.1360-0443.2008.02277.x 18855814 PMC2690607

[13] Verster JC, Roth T (2011) Standard operation procedures for conducting the on-the-road driving test, and measurement of the standard deviation of lateral position (SDLP). Int J Gen Med 4: 359–371. 10.2147/IJGM.S19639 21625472 PMC3100218

[14] Borkenstein F, Crowther RF, Shumate RP, Zeil WB, Zylman R (1964) The role of the drinking driver in traffic accidents. Indiana University, Bloomington.

[15] Schnabel E, Hargutt V, Krüger, H (2010) Meta-analysis of empirical studies concerning the effects of alcohol on safe driving. Deliverable 112a, DRUID (Driving under the Influence of Drugs, Alcohol and Medicines) 6th framework programme. Available at http://www.druid-project.eu/. Accessed 10 December 2013.

[16] Brookhuis KA, Waard DD, Fairclough SH (2003) Criteria for driver impairment. Ergonomics 46(5): 433–445. 12745694 10.1080/001401302/1000039556

[17] Van Steveninck AL, Van Berckel BNM, Schoemaker RC, Breimer DD, Van Gerven et al (1999) The sensitivity of pharmacodynamic tests for the central nervous system effects of drugs on the effects of sleep deprivation. Journal of Psychopharmacology 13(1): 10–17. 10221355 10.1177/026988119901300102

[18] Connor J, Norton R, Ameratunga S, Robinson E, Civil I, et al (2002) Driver sleepiness and risk of serious injury to car occupants: population based case control study. BMJ 324(7346): 1125 12003884 10.1136/bmj.324.7346.1125 PMC107904

[19] Horne JA, Reyner LA (1995) Sleep related vehicle accidents. BMJ 310(6979): 565–567. 7888930 10.1136/bmj.310.6979.565 PMC2548939

[20] Akerstedt T, Kecklund G, Hörte LG (2001) Night driving, season, and the risk of highway accidents. Sleep 24(4): 401–406. 11403524 10.1093/sleep/24.4.401

[21] Martiniuk AL, Senserrick T, Lo S, Williamson A, Du W, et al (2013) Sleep-deprived young drivers and the risk for crash: the DRIVE prospective cohort study. JAMA pediatr 167(7): 647–655. 10.1001/jamapediatrics.2013.1429 23689363

[22] Valent F, Di Bartolomeo S, Marchetti R, Sbrojavacca R, Barbone F (2010) A case-crossover study of sleep and work hours and the risk of road traffic accidents. Sleep 33(3): 349 20337193 10.1093/sleep/33.3.349 PMC2831429

[23] Baulk SD, Biggs SN, Reid KJ, van den Heuvel CJ, Dawson D (2008) Chasing the silver bullet: measuring driver fatigue using simple and complex tasks. Accid Anal Prev 40(1): 396–402. 10.1016/j.aap.2007.07.008 18215574

[24] Bosker WM, Kuypers KP, Conen S, Ramaekers JG (2010) Dose-related effects of MDMA on psychomotor function and mood before, during, and after a night of sleep loss. Psychopharmacology 209(1): 69–76. 10.1007/s00213-009-1767-1 20084368 PMC2819659

[25] Gillberg M, Åkerstedt T (1998) Sleep loss and performance: no “safe” duration of a monotonous task. Physiology & behavior 64(5): 599–604. 10.1016/j.domaniend.2014.12.002 9817569

[26] Jackson ML, Croft RJ, Kennedy GA, Owens K, Howard ME (2013) Cognitive components of simulated driving performance: sleep loss effects and predictors. Accid Anal Prev 50: 438–444. 10.1016/j.aap.2012.05.020 22721550

[27] Kuypers KPC, Wingen M, Samyn N, Limbert N, Ramaekers JG (2007) Acute effects of nocturnal doses of MDMA on measures of impulsivity and psychomotor performance throughout the night. Psychopharmacology 192(1): 111–9. 17219216 10.1007/s00213-006-0679-6

[28] Lamond N, Dawson D (1999) Quantifying the performance impairment associated with fatigue. J Sleep Res 8(4): 255–262. 10646165 10.1046/j.1365-2869.1999.00167.x

[29] Williamson AM, Feyer AM (2000) Moderate sleep deprivation produces impairments in cognitive and motor performance equivalent to legally prescribed levels of alcohol intoxication. Occup Environ Med 57(10): 649–655. 10984335 10.1136/oem.57.10.649 PMC1739867

[30] Doran S (2001) Sustained attention performance during sleep deprivation: evidence of state instability. Arch Ital Biol 139(3): 253–267. 11330205

[31] Jewett ME, Dijk DJ, Kronauer RE, Dinges DF (1999) Dose-response relationship between sleep duration and human psychomotor vigilance and subjective alertness. Sleep 22(2): 171–179. 10201061 10.1093/sleep/22.2.171

[32] Lim J, Dinges DF (2008) Sleep deprivation and vigilant attention. Annals of the New York Academy of Sciences 1129(1): 305–322. 18591490 10.1196/annals.1417.002

[33] Schuhfried G (2005) Manual Expert System Traffic (XPSV). Mödling: Schuhfried GmbH

[34] Brunnauer A, Laux G, David I, Fric M, Hermisson I, et al (2008) The impact of reboxetine and mirtazapine on driving simulator performance and psychomotor function in depressed patients. J Clin Psychiatry 69(12): 1880–1886. 19203476 10.4088/jcp.v69n1205

[35] Greenblatt DJ, Legangneux E, Harmatz JS, Weinling E, Freeman J, et al (2006) Dynamics and kinetics of a modified‐release formulation of zolpidem: comparison with immediate‐release standard zolpidem and placebo. J Clin Pharmacol 46(12): 1469–1480. 17101746 10.1177/0091270006293303

[36] Moskowitz H (1973) Laboratory studies of the effects of alcohol on some variables related to driving J Sleep Res 1973(5): 185–199.

[37] Robbe HW, O’Hanlon JF (1995) Acute and subchronic effects of paroxetine 20 and 40 mg on actual driving, psychomotor performance and subjective assessments in healthy volunteers. Europ Neuropsychopharmacol 5(1) 35–42. 10.1016/0924-977x(94)00130-4 7613099

[38] Roth T, Mayleben D, Corser BC, Singh NN (2008) Daytime pharmacodynamic and pharmacokinetic evaluation of low‐dose sublingual transmucosal zolpidem hemitartrate. Hum Psychopharmacol 23(1): 13–20. 17907263 10.1002/hup.884 PMC2871168

[39] Forsman P, Pyykkö I, Toppila E, Hæggström E (2014) Feasibility of force platform based roadside drowsiness screening—A pilot study. Accid Anal Prev 62: 186–190. 10.1016/j.aap.2013.09.015 24172085

[40] Jongen S, Vuurman E, Ramaekers J, Vermeeren A. (2014) Alcohol calibration of tests measuring skills related to car driving. Psychopharmacology 231(12): 2435–2447. 10.1007/s00213-013-3408-y 24408210 PMC4039994

[41] Clark PG, Blissmer BJ, Greene GW, Lees FD, Riebe DA, et al (2011) Maintaining exercise and healthful eating in older adults: The SENIOR project II: Study design and methodology. Contemp Clin Trials 32(1): 129–139. 10.1016/j.cct.2010.10.002 20955821 PMC4533928

[42] Silva MT, Laks J, Engelhardt E (2009) Neuropsychological tests and driving in dementia: a review of the recent literature. Revista da Associação Médica Brasileira 55(4): 484–488. 19750319 10.1590/s0104-42302009000400027

[43] Myers RS, Ball KK, Kalina TD, Roth DL, Goode KT (2000) Relation of useful field of view and other screening tests to on-road driving performance. Percept Mot Skills 91(1): 279–90. 11011899 10.2466/pms.2000.91.1.279

[44] Weaver B, Bédard M, McAuliffe J, Parkkari M (2009) Using the Attention Network Test to predict driving test scores. Accid Anal Prev 41(1): 76–83. 10.1016/j.aap.2008.09.006 19114140

[45] Louwerens J, Gloerich A, de Vries G, Brookhuis K, O’Hanlon J (1987) The relationship between drivers’ blood alcohol concentration (BAC) and actual driving performance during high speed travel In: Noordzij PC, Roszbach R, editors. Alcohol Drugs Traffic Safety—T86. Amsterdam: Elsevier pp. 183–186. 10.1080/15389588.2014.935356

[46] Vermeeren A, Riedel WJ, Van Boxtel MP, Darwish M, Paty I, et al (2002) Differential residual effects of zaleplon and zopiclone on actual driving: a comparison with a low dose of alcohol. Sleep 25(2): 224–231. 11905433

[47] O’Hanlon JF, Ramaekers JG (1995) Antihistamine effects on actual driving performance in a standard test: a summary of Dutch experience, 1989‐94. Allergy 50(3): 234–242. 7677241 10.1111/j.1398-9995.1995.tb01140.x

[48] Vuurman EFPM, Rikken GH, Muntjewerff ND, De Halleux F, Ramaekers JG (2004) Effects of desloratadine, diphenhydramine, and placebo on driving performance and psychomotor performance measurements. European J Clin Pharmacol 60(5): 307–313. 15168099 10.1007/s00228-004-0757-9

[49] Leufkens TR, Vermeeren A (2014) Zopiclone’s Residual Effects on Actual Driving Performance in a Standardized Test: A Pooled Analysis of Age and Sex Effects in 4 Placebo-Controlled Studies. Clinical therapeutics 36(1): 141–150. 10.1016/j.clinthera.2013.11.005 24360801

[50] Roth T, Eklov SD, Drake CL, Verster JC (2014) Meta-analysis of on-the-road experimental studies of hypnotics: effects of time after intake, dose, and half-life. Traffic Inj Prev 15(5): 439–445. 10.1080/15389588.2013.830211 24678565

[51] Vermeeren A, Vuurman EF, Leufkens TR, Van Leeuwen CJ, Van Oers AC et al (2013) Residual Effects of Low-Dose Sublingual Zolpidem on Highway Driving Performance the Morning after Middle-of-the-Night Use. Sleep 37(3): 489–496. 10.5665/sleep.3482 PMC3920314 24587571

[52] Bosker WM, Kuypers KPC, Conen S, Kauert GF, Toennes SW, et al (2012) MDMA (ecstasy) effects on actual driving performance before and after sleep deprivation, as function of dose and concentration in blood and oral fluid. Psychopharmacology 222(3): 367–76. 10.1007/s00213-011-2497-8 21952668 PMC3395348

[53] Verster JC, Roth T (2012) Predicting psychopharmacological drug effects on actual driving performance (SDLP) from psychometric tests measuring driving-related skills. Psychopharmacology 220(2): 293–301. 10.1007/s00213-011-2484-0 21922169 PMC3285752

[54] Mulder-Hajonides van der Meulen WREH, Van den Hoofdakker RH (1984). The Groningen sleep quality scale. In: Book of Abstracts 14th CINP Congress Florence. 25057717

[55] Dinges D, Powell J (1985) Microcomputer analyses of performance on a portable, simple visual RT task during sustained operations. Behav Res Methods 17(6): 652–655.

[56] Basner M, Dinges DF (2011) Maximizing sensitivity of the psychomotor vigilance test (PVT) to sleep loss. Sleep 34(5): 581 21532951 10.1093/sleep/34.5.581 PMC3079937

[57] Jex HR, McDonnell JD, Phatak AV (1966) A “critical” tracking task for man-machine research related to the operator’s effective delay time. I. Theory and experiments with a first-order divergent controlled element. NASA CR-616. NASA contractor report. NASA CR. United States. National Aeronautics and Space Administration, 1–105. 5297174

[58] Riedel WJ, Mehta MA, Unema PJ (2006) Human cognition assessment in drug research. Current pharmaceutical design 12(20): 2525–2539. 16842175 10.2174/138161206777698882

[59] McLeod DR, Griffiths RR, Bigelow GE, Yingling J (1982) An automated version of the digit symbol substitution test (DSST). Behav Res Methods Instrum Comput 14(5): 463–466.

[60] Leufkens TRM, Lund JS, Vermeeren A (2009) Highway driving performance and cognitive functioning the morning after bedtime and middle-of-the-night use of gaboxadol, zopiclone and zolpidem. J Sleep Res 18(4): 387–96. 10.1111/j.1365-2869.2009.00746.x 19552733

[61] Fan J, McCandliss BD, Sommer T, Raz A, Posner MI (2002) Testing the efficiency and independence of attentional networks. Journal of cognitive neuroscience 14(3): 340–347. 11970796 10.1162/089892902317361886

[62] Van der Elst W, Van Boxtel MP, Van Breukelen GJ, Jolles J (2006) The Concept Shifting Test: adult normative data. Psychological Assessment 18: 424–432. 17154763 10.1037/1040-3590.18.4.424

[63] Mets MAJ, Volkerts ER, Olivier B, Verster JC (2010) Effect of hypnotic drugs on body balance and standing steadiness. Sleep Med Rev 14(4): 259–267. 10.1016/j.smrv.2009.10.008 20171127

[64] Mets MAJ, de Vries JM, de Senerpont Domis LM, Volkerts ER, Olivier B, et al (2011) Next-day effects of ramelteon (8 mg), zopiclone (7.5 mg), and placebo on highway driving performance, memory functioning, psychomotor performance, and mood in healthy adult subjects. Sleep 34(10): 1327–1334. 10.5665/SLEEP.1272 21966064 PMC3173578

[65] Edwards JD, Vance DE, Wadley VG, Cissell GM, Roenker DL, et al (2005) Reliability and validity of useful field of view test scores as administered by personal computer. Journal of Clinical and Experimental Neuropsychology 27(5): 529–543. 16019630 10.1080/13803390490515432

[66] Åkerstedt T, Gillberg M (1990) Subjective and objective sleepiness in the active individual. International Journal of Neuroscience 52(1–2): 29–37. 2265922 10.3109/00207459008994241

[67] Brookhuis KA, de Vries G, O’Hanlon JF (1985) The effects of increasing doses of meptazinol (100, 200, 400 mg) and glafenine (200 mg) on actual driving performance VK 85–16. Haren: Traffic Research Centre.

[68] Dorrian J, Rogers NL, Dinges DF (2005) Psychomotor vigilance performance: Neurocognitive assay sensitive to sleep loss. PhD Thesis, University of Pensylvania School of Medicine. Available: https://www.med.upenn.edu/uep/user_documents/Dorrianetal.PVTchapterinKushida2005.pdf. Accessed 10 June 2012.

[69] Dry MJ, Burns NR, Nettelbeck T, Farquharson AL, White JM (2012) Dose-related effects of alcohol on cognitive functioning. PloS one 7(11): e50977 10.1371/journal.pone.0050977 23209840 PMC3510176

[70] Dunlap WP, Cortina JM, Vaslow JB, Burke MJ (1996) Meta-analysis of experiments with matched groups or repeated measures designs. Psychological Methods 1(2): 170–177.

[71] Falleti MG, Maruff P, Collie A, Darby DG, McStephen M (2003) Qualitative similarities in cognitive impairment associated with 24 h of sustained wakefulness and a blood alcohol concentration of 0.05%. J Sleep Res 12(4): 265–274. 14633237 10.1111/j.1365-2869.2003.00363.x

[72] Boyle J, Danjou P, Alexander R, Calder N, Gargano C, et al (2009) Tolerability, pharmacokinetics and night‐time effects on postural sway and critical flicker fusion of gaboxadol and zolpidem in elderly subjects. British journal of clinical pharmacology 67(2): 180–190. 10.1111/j.1365-2125.2008.03331.x 19094161 PMC2670375

[73] Howard ME, Jackson ML, Kennedy GA, Swann P, Barnes M, et al (2007) The interactive effects of extended wakefulness and low-dose alcohol on simulated driving and vigilance. Sleep 30(10): 1334 17969467 10.1093/sleep/30.10.1334 PMC2266271

[74] Leufkens TRM, Ramaekers JG, de Weerd AW, Riedel WJ, Vermeeren A. (2014) Residual effects of zopiclone 7.5 mg on highway driving performance in insomnia patients and healthy controls: a placebo controlled crossover study. Psychopharmacology: 1–14. 10.1007/s00213-014-3807-8 24458443 PMC4072058

[75] Rogé J, Pébayle T, Hannachi SE, Muzet A (2003) Effect of sleep deprivation and driving duration on the useful visual field in younger and older subjects during simulator driving. Vision research 43(13): 1465–1472. 12767314 10.1016/s0042-6989(03)00143-3

[76] Jugovac D, Cavallero C (2012) Twenty-four hours of total sleep deprivation selectively impairs attentional networks. Experimental psychology 59(3): 115–23. 10.1027/1618-3169/a000133 22044791

[77] Martella D, Casagrande M, Lupiáñez J (2011) Alerting, orienting and executive control: the effects of sleep deprivation on attentional networks. Experimental brain research 210(1): 81–89. 10.1007/s00221-011-2605-3 21390488

[78] Dawson D, Reid K (1997) Fatigue, alcohol and performance impairment. Nature, 388(6639): 235 9230429 10.1038/40775

[79] Borbély AA (1982) A two process model of sleep regulation. Hum Neurobiol. 7185792

[80] Owens K, Ramaekers JG (2009) Drugs, driving, and models to measure driving impairment In Verster JC, Pandi-Perumal SR, Ramaekers JG, de Gier JJ, editors. Drugs, driving and traffic safety. Basel: Birkhäuser pp. 43–58

[81] Kleykamp BA, Griffiths RR, Mintzer MZ (2010) Dose effects of triazolam and alcohol on cognitive performance in healthy volunteers. Exp Clin Psychopharmacol 18(1): 1 10.1037/a0018407 20158290 PMC2847582

[82] Tiplady B, Hiroz J, Holmes L, Drummond G (2003) Errors in performance testing: a comparison of ethanol and temazepam. Journal of Psychopharmacology 17(1): 41–49. 12680738 10.1177/0269881103017001691

